# Consumer Devices for Patient-Generated Health Data Using Blood Pressure Monitors for Managing Hypertension: Systematic Review

**DOI:** 10.2196/33261

**Published:** 2022-05-02

**Authors:** Jonathan R Treadwell, Benjamin Rouse, James Reston, Joann Fontanarosa, Neha Patel, Nikhil K Mull

**Affiliations:** 1 ECRI Plymouth Meeting, PA United States; 2 Division of General Internal Medicine Department of Medicine Perelman School of Medicine, University of Pennsylvania Philadelphia, PA United States; 3 Center for Evidence-based Practice University of Pennsylvania Health System Philadelphia, PA United States

**Keywords:** patient-generated health data, consumer devices, hypertension, blood pressure monitors, digital health, cardiovascular diseases, wearable devices, health information, mobile phone

## Abstract

**Background:**

In the era of digital health information technology, there has been a proliferation of devices that collect patient-generated health data (PGHD), including consumer blood pressure (BP) monitors. Despite their widespread use, it remains unclear whether such devices can improve health outcomes.

**Objective:**

We performed a systematic review of the literature on consumer BP monitors that collect PGHD for managing hypertension to summarize their clinical impact on health and surrogate outcomes. We focused particularly on studies designed to measure the specific effect of using a BP monitor independent of cointerventions. We have also summarized the process and consumer experience outcomes.

**Methods:**

An information specialist searched PubMed, MEDLINE, and Embase for controlled studies on consumer BP monitors published up to May 12, 2020. We assessed the risk of bias using an adapted 9-item appraisal tool and performed a narrative synthesis of the results.

**Results:**

We identified 41 different types of BP monitors used in 49 studies included for review. Device engineers judged that 38 (92%) of those devices were similar to the currently available consumer BP monitors. The median sample size was 222 (IQR 101-416) participants, and the median length of follow-up was 6 (IQR 3-12) months. Of the included studies, 18 (36%) were designed to isolate the clinical effects of BP monitors; 6 of the 18 (33%) studies evaluated health outcomes (eg, mortality, hospitalizations, and quality of life), and data on those outcomes were unclear. The lack of clarity was due to low event rates, short follow-up duration, and risk of bias. All 18 studies that isolated the effect of BP monitors measured both systolic and diastolic BP and generally demonstrated a decrease of 2 to 4 mm Hg in systolic BP and 1 to 3 mm Hg in diastolic BP compared with non–BP monitor groups. Adherence to using consumer BP monitors ranged from 38% to 89%, and ease of use and satisfaction ratings were generally high. Adverse events were infrequent, but there were a few technical problems with devices (eg, incorrect device alerts).

**Conclusions:**

Overall, BP monitors offer small benefits in terms of BP reduction; however, the health impact of these devices continues to remain unclear. Future studies are needed to examine the effectiveness of BP monitors that transmit data to health care providers. Additional data from implementation studies may help determine which components are critical for sustained BP improvement, which in turn may improve prescription decisions by clinicians and coverage decisions by policy makers.

## Introduction

In 2018, nearly half a million deaths in the United States included hypertension as a primary or contributing cause [1]. Current data support the use of out-of-office blood pressure (BP) monitoring for hypertension management because it provides clinical information beyond in-office BP monitoring and enhances titration of the medication dose [2-4]. This evidence has led to the proliferation of consumer patient-generated health data (PGHD) devices for hypertension management.

The Office of the National Coordinator for Health Information Technology defines PGHD as “health-related data created, recorded, or gathered by or from patients (or family members or other caregivers) to help address a health concern” [5]. These health-related data are captured by the patient, who may also need to share this information with a health care provider or others (if data transmission is not automatic). The adoption curve of consumer PGHD devices for hypertension management is maturing due to the rising numbers of wearables and BP monitors on the market. The global market size of automated home BP monitors is expected to gain market growth between 2020 and 2025, with a compound annual growth rate of 2.3%, forecasting US $1068.3 million by 2025, from US $975.6 million in 2019 [6].

Consumer PGHD devices can improve the health outcomes of patients and play an important role in managing hypertension. This review summarizes findings on hypertension from a larger report that addressed PGHD for 11 chronic conditions. The full report can be downloaded from the website of the Effective Healthcare Program at the Agency for Healthcare Research and Quality (AHRQ) [7]. In this paper, we summarize the clinical effectiveness of consumer BP monitors in collecting PGHD on health and surrogate outcomes. We also summarize the process outcomes (eg, medication titration) and consumer experience outcomes (eg, device adherence, ease of use, and technical problems).

## Methods

### Search Strategy

A professional information specialist searched MEDLINE and Embase, in-process MEDLINE and PubMed unique content, and the Cochrane Database of Systematic Reviews for systematic reviews or controlled trials published from inception until May 12, 2020. We also searched ClinicalTrials.gov for active studies until June 19, 2020. The review protocol is posted on the PROSPERO website [7].

### Selection Criteria

Textbox 1 shows study eligibility criteria for studies evaluating the effects of BP monitors on hypertension. Device engineers examined the devices from the screened studies (manufacturer and model names) and determined whether each device was available for direct purchase by consumers. Studies that included nonconsumer devices (eg, devices requiring a prescription) were excluded. The technology had to collect and store consumer data without requiring manual input and potentially could be sent to a health care professional, although data transmission was not required for study inclusion. We included both US-marketed and non–US-marketed technologies that met the criteria. However, any technology subject to Food and Drug Administration (FDA) clearance must have received FDA clearance to be included.

We carefully examined the interventions provided to each treatment group and determined whether the study design isolated the effect of the BP monitor. This occurred when the intervention group received the BP monitor whereas other comparison groups did not, and any additional treatments were the same between groups. In cases where clinicians made changes to treatment plans (eg, medication or dose adjustments) based on feedback from the BP monitor, we considered it as part of the BP monitor’s effect because such adjustments were only possible due to the device. The comparison groups commonly received usual care, which would not preclude the clinician’s decisions to modify hypertension treatment plans based on BP measurements in other contexts and settings.

Using DistillerSR (Evidence Partners), 3 reviewers (JRT, BR, and JR) screened the titles, and all 6 screened abstracts and full-text articles. For titles, only 1 reviewer assessed the general relevance to the topic. For abstract screening, 2 reviewers were necessary to exclude an article from further consideration; however, only 1 reviewer was necessary to order the full text. Regarding full texts, 2 reviewers assessed the study against the inclusion criteria, and disagreements were resolved by a (senior-level) third reviewer (JRT or JR). Full-text screening also involved determining which articles were associated with other included articles of the same trial.

**Eligibility criteria.**

**Category and criteria**

- Populations
  Include individuals who have (or may potentially develop) hypertension
  Exclude individuals with other conditions and pregnant and postpartum women
- Interventions
  Include consumer blood pressure (BP) monitors for the prevention or treatment of hypertension. The monitor must collect and store the patient data without manual input, which could be used by the patient or sent to a health care professional (data transmission was not required but could be via the same or a different technology)
- Comparators
  Include non–patient-generated health data (PGHD) interventions, other PGHD interventions, or no intervention
  Exclude comparators that used the same PGHD intervention
- Outcomes
  Include health outcomes: direct measures of health (eg, mortality, emergency room visits, hospitalizations, disease progression, and quality of life)
  Include blood pressure: systolic or diastolic BP change and change in BP control
  Include potential harms: serious adverse events (eg, hospitalization or delay in care) and other potential harms such as underuse or overuse of medications secondary to inaccurate BP data
  Include process outcomes (if 1 of the first 3 outcome categories were reported): medication changes
  Include consumer outcomes (if 1 of the first 3 outcome categories were reported): BP measurement adherence, interoperability, functions, acceptability/usability, sustainability, feasibility, fidelity, and integration into electronic health records
  Include costs (if 1 of the first 3 outcome categories were reported): total cost and cost-effectiveness
  Exclude surrogates such as prescription filling behavior, biomarkers that do not define the condition, adherence, disease knowledge, beliefs, opinions, dietary behavior, activity level, and steps per day
- Timing/setting
  Include no limitations on timing. The setting must be at home or otherwise outside of a hospital or health care center.
- Study designs
  Include any study design with a separate comparison group of patients who received a different intervention strategy or single-arm registry studies. Systematic reviews were only used to screen their included studies to ensure none were missed by the database searches.
  Exclude reviews, case reports, editorials, comments, letters, meeting abstracts, and studies with <10 patients per arm at follow-up.
- Language
  Include studies published in English.

### Data Extraction

For each included trial, 1 reviewer (BR or NM) extracted the general trial information, patient characteristics (eg, baseline BP), treatment details (including specific PGHD devices), risk-of-bias items, and outcome data. We examined data on the following reported health outcomes: mortality, emergency room visits, hospitalization, quality of life (QoL), and adverse events (AEs). Surrogate outcomes for hypertension consisted of systolic BP (SBP) and diastolic BP (DBP). Process outcomes included medication changes, dose adjustments, physician consultations, and office visits. We also extracted data on consumer experience, including device adherence, the number of BP readings taken or transmitted, device alerts, ease of use, patient satisfaction, and technical problems.

### Risk-of-Bias Assessment

We assessed the overall risk of bias based on 9 items, including randomization, allocation concealment, baseline similarity between groups, and masking of outcome assessors. The items were adapted from the AHRQ report titled “Mobile Applications for Self-Management of Diabetes” [8]. In addition, we included an item about whether the device’s effects could be isolated (ie, consumer BP monitor alone vs usual care). After considering all 9 items, we categorized each trial as at low, moderate, or high risk of bias.

### Device Similarity

Given that the included studies were published as early as 1997, for each BP monitor used within the included studies, device engineers assessed the similarity to devices currently on the market from that manufacturer. They used the following scale: (1) this model is *similar* to a device available from this manufacturer; (2) this model is *somewhat different* than any device available from this manufacturer; (3) this model is *very different* from any device available from this manufacturer; and (4) we could not reliably determine the similarity of this model with the ones currently available from this manufacturer.

### Results Classification

For isolated effects on health outcomes, we narratively synthesized the summary effect into one of four categories: (1) likely no effect, (2) unclear, (3) possible positive effect, or (4) likely positive effect. If the results consistently demonstrated the lack of an effect (via narrow CIs around a null effect), we coded it as likely no effect. If the results were inconsistent in the direction of effect or study authors could not reach a conclusion, the findings were coded as *unclear* for that outcome. If ≥1 outcomes had minor inconsistency in findings, but at least 1 study with moderate or low risk of bias showed a positive effect, the findings were coded as *possible positive effect*. If the results had a consistent positive effect, we coded it as *likely positive effect*.

When we categorized health outcome data as *unclear*, we then examined surrogate outcomes, which for hypertension were SBP and DBP. To help interpret the SBP/DBP outcomes, we used a minimally important difference of 2 mm Hg [9,10].

For studies of multicomponent interventions, we did not attempt to classify the data in the manner described earlier because the effect of BP monitoring in those studies could not be determined.

## Results

### Literature Search

**Figure 1:**
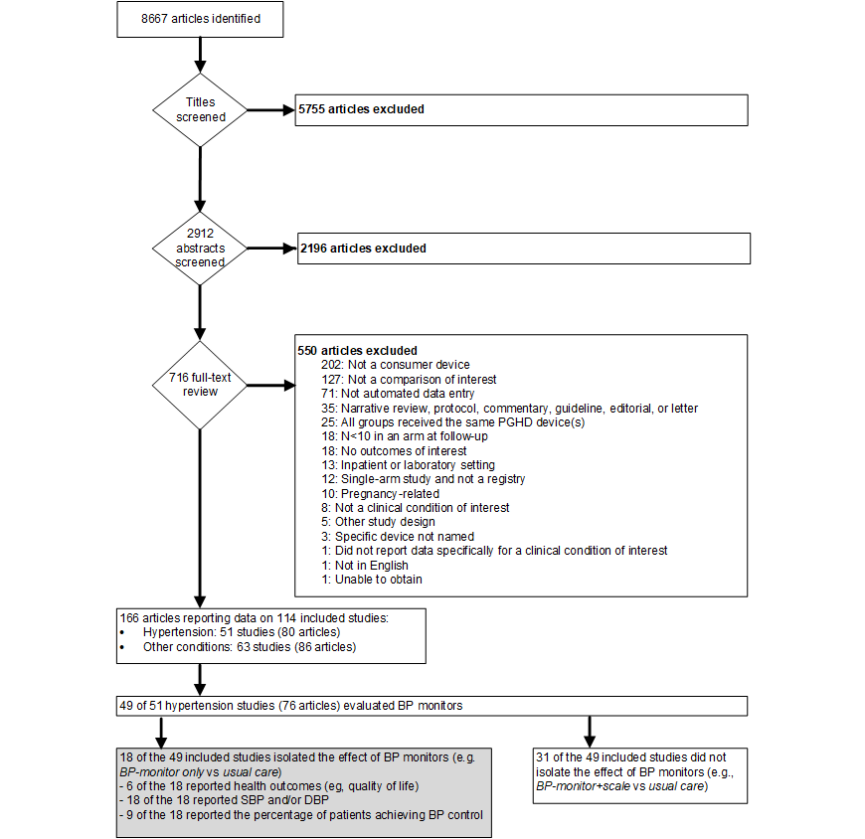
Study flow diagram. BP: blood pressure; DBP: diastolic blood pressure; PGHD: patient-generated health data; SBP: systolic blood pressure.

For the full report (ie, 11 clinical conditions), our searches identified 8667 potentially relevant articles, of which we excluded 5755 (66.40%) at the title level (not relevant) and 2196 (25.33%) at the abstract level (Figure 1). We dual-screened the full texts of the remaining 716 articles (8.26%). The review team included 126 (17.6%) of these studies, but upon further review of the devices by device engineers, 12 studies (1.7%) had used only nonconsumer devices and were therefore excluded from the full report (none of the 12 addressed hypertension). A total of 114 unique studies were described in 166 articles. For the subset of screened studies enrolling patients with hypertension, we included 51 studies reported in 80 articles. This review focuses on 49 (96%; 79 articles) of those 51 studies that used BP monitors to generate PGHD for managing hypertension; 2 studies did not use BP monitors to manage hypertension, 1 evaluated a pedometer [11], and the other compared 2 mobile apps [12]. Of the 49 studies, 18 (36.7%) used designs that isolated the effect of BP monitors (eg, BP monitor alone vs usual care or BP monitor+scale vs scale alone), whereas the other 31 (63.3%) used multicomponent designs that did not permit conclusions about the impact on outcomes specific to BP monitors (eg, BP monitor+scale vs usual care).

### Study Characteristics

Key characteristics of the studies using BP monitors for hypertension are shown in Table 1 (18 isolated-effect studies) and Multimedia Appendix 1 (Table S1; 31 multicomponent studies). Of the 49 studies, 47 (96%) were randomized trials, and 2 (4%) were nonrandomized; 21 (43%) studies were conducted in the United States, and other notable countries included the United Kingdom (n=6 studies, 12%), Canada (n=3 studies, 6%), Denmark (n=2 studies. 4%), Finland (n=2 studies, 4%), and South Korea (n=2 studies, 4%). The median number of patients per study at baseline was 222 (IQR 80-433). Patient enrollment dates were reported in 29 (59%) studies and ranged from May 1999 to June 2017. The median length of follow-up was 6 months (IQR 4-12).

Study group comparisons are shown in Table 2 and Multimedia Appendix 1 (Table S1). Of the 49 studies, 42 (86%) had 2 study groups, 4 (8%) studies had 3 groups, and 3 (6%) studies had 4 groups. A usual care control group was used in 43 (88%) studies, whereas 3 (6%) studies used a consumer device in the control group, and 4 (8%) other studies used active comparators without a consumer device (eg, counseling alone). Statistical power analyses were conducted a priori in 39 of the 49 (80%) studies, and 29 of these 39 (74%) studies were based on SBP, DBP, or BP control. Note that 31 of the 49 (62%) studies used only multicomponent interventions, making it impossible to discern the impact specific to the BP monitor. Among these 31 studies, 25 (81%) used a BP monitor along with nondevice interventions, 3 (10%) studies used a BP monitor along with another device, and the other 4 (12%) studies used a BP monitor along with ≥2 other devices.

**Table 1:** General characteristics of studies isolating the effect of blood pressure monitors.

Study	Design	Country	N at baseline	Study duration	Study groups (BP^a^ monitor manufacturer and model)	Outcomes
Aekplakorn et al (2016) [13]	RCT^b^	Thailand	224	12 months	PGHD^c^ (Omron HEM 7117) Usual care	Surrogate (SBP^d^, DBP^e^, or BP control) Process Consumer experience
Bosworth et al (2009) [14]	RCT	United States	636	2 years	PGHD (Omron 773AC or 637) Behavioral intervention Combination (PGHD+behavioral) Usual care	Health (hospitalizations) Surrogate (SBP, DBP, or BP control) Process Adverse events Consumer experience
Bosworth et al (2011) [15-17]	RCT	United States	636	24 months	PGHD (Omron 773AC or 637) Behavioral intervention Combination (PGHD+behavioral) Usual care	Surrogate (SBP or DBP)
Broege 2001 [18]	RCT	United States	40	3 months	PGHD (Omron HEM-702) Usual care	Health (QoL^f^) Surrogate (SBP or DBP) Consumer experience
Fuchs et al (2012) [19]	RCT	Brazil	121	60 days	PGHD (Omron HEM-705 CP) Usual care	Surrogate (SBP or DBP) Consumer experience
Green et al (2008) [20,21]	RCT	United States	778	1 year	PGHD (Omron HEM-705 CP) Combination (PGHD+pharmacist care) Usual care	Health (QoL) Surrogate (SBP, DBP, or BP control) Adverse events
Hebert et al (2012) [22]	RCT	United States	416	18 months	PGHD (Omron HEM-712C) Combination (PGHD+nurse management) Usual care	Health (mortality) Surrogate (SBP, DBP, or BP control) Process
Hoffmann-Petersen et al (2017) [23]	RCT	Denmark	356	3 months	PGHD (A&D 767PlusBT or Omron 705IT) Usual care	Surrogate (SBP, DBP, or BP control) Process
Hosseininasab et al (2014) [24]	RCT	Iran	194	24 weeks	PGHD (Samsung SHB-200w) Usual care	Surrogate (SBP or DBP)
Kaihara et al (2014) [25]	RCT	Japan	57	2 weeks	PGHD (Omron HEM-7251G) Conventional BP monitor	Surrogate (SBP or DBP) Consumer experience
Kauric-Klein et al (2007) [26]	RCT	United States	34	12 weeks	PGHD (Omron IC) Usual care	Surrogate (SBP or DBP)
Kim et al (2016) [27,28]	RCT	United States	160	6 months	PGHD (Withings) Usual care	Surrogate (SBP, DBP, or BP control) Consumer experience
Lakshminarayan et al (2018) [29]	RCT	United States	50	13 weeks	PGHD (upper arm Withings [Nikia] wireless BP monitor) Conventional BP monitor	Surrogate (SBP) Consumer experience
Márquez-Contreras et al (2006) [30]	RCT	Spain	250	6 months	PGHD (Omron M4 automatic monitor) Usual care	Surrogate (SBP, DBP, or BP control)
McManus et al (2018) [4,31-33]	RCT	United Kingdom	1173	12 months	PGHD (Omron M10-IT) Combination (PGHD+telemonitoring) Usual care	Health (QoL) Surrogate (SBP or DBP); process Adverse events
Qi et al (2017) [34]	RCT	China	1032	5 years	PGHD (Omron HEM-7121) Control group	Surrogate (SBP, DBP, or BP control)
Zaleski et al (2019) [35]	RCT	United States	24	4 months	PGHD (BP Omron 705 CPN) Usual care	Surrogate (SBP or DBP) Adverse events Consumer experience
Zha et al (2019) [36]	RCT	United States	25	6 months	PGHD (iHealth BP 7 wireless BP wrist monitor) Usual care	Health (QoL) Surrogate (SBP, DBP, or BP control) Consumer experience

^a^BP: blood pressure.

^b^RCT: randomized controlled trial.

^c^PGHD: patient-generated health data.

^d^SBP: systolic blood pressure.

^e^DBP: diastolic blood pressure.

^f^QoL: quality of life.

**Table 2:** Patient characteristics in studies isolating the effect of blood pressure monitors.

Study	Age (years), mean	Sample (female), n	Female, n (%)	Baseline disease severity
Aekplakorn et al (2016) [13]	59	224	148 (66)	Mean SBP^a^ PGHD^b^: 149.4 mm Hg Mean DBP^c^ PGHD: 83.4 mm Hg Mean SBP UC^d^: 147.2 mm Hg Mean DBP UC: 82.2 mm Hg
Bosworth et al (2009) [14]	61	636	420 (66)	BP controlled at baseline 73% Mean SBP: 125 mm Hg Mean DBP: 71 mm Hg
Bosworth et al (2011) [15]; Bosworth et al (2007) [16]; Bosworth et al (2008) [17]	61	636	407 (64)	Mean SBP: 125 mm Hg Mean DBP: 71 mm Hg
Broege et al (2001) [18]	73	40	28 (70)	Mean ambulatory awake SBP: 147 mm Hg Mean ambulatory awake DBP: 82 mm Hg
Fuchs et al (2012) [19]	59.0	121	73 (60)	Mean office SBP: 158.6 mm Hg Mean office DBP: 89.5 mm Hg Mean 24-hour systolic ABPM^e^: 148.8 mm Hg Mean 24-hour diastolic ABPM: 87.5 mm Hg
Green et al (2008) [20,21]	59.1	778	405 (52)	Mean SBP: 151.9 mm Hg Mean DBP: 89.1 mm Hg
Hebert et al (2012) [22]	60.8	416	295 (71)	Mean SBP: 153 mm Hg Mean DBP: 86.0 mm Hg
Hoffmann-Petersen et al (2017) [23]	60.5	356	164 (46)	Mean office SBP: 154.6 mm Hg Mean office DBP: 93.2 mm Hg
Hosseininasab et al (2014) [24]	58.7	194	118 (61)	Mean SBP: 145.2 mm Hg Mean DBP: 85.3 mm Hg
Kaihara et al (2014) [25]	64.4	57	37 (65)	Mean SBP: 144 mm Hg Mean DBP: 83 mm Hg
Kauric-Kleinet et al (2007) [26]	48.7	34	23 (68)	Mean SBP PGHD: 161 mm Hg and 162 mm Hg in the UC group Mean DBP PGHD: 94 mm Hg Mean DBP UC: 100 mm Hg Patients were chronic hemodialysis patients
Kim et al (2016) [27]; Bloss (2016) [28]	57.6	160	104 (65)	Mean SBP: 140.6 mm Hg Mean DBP: 89.4 mm Hg Mean number of antihypertensive medications: 2
Lakshminarayan et al (2018) [29]	66	50	14 (28)	Mean SBP: 140 mm Hg Mean DBP: not reported
Márquez-Contreras et al (2006) [30]	59.1	250	123 (49)	Mean SBP: 157.4 mm Hg Mean DBP: 91.7 mm Hg
McManus et al (2018) [4,31-33]	66.9	1173	540 (46)	Mean SBP: 153.1 mm Hg Mean DBP: 85.5 mm Hg
Qi et al (2017) [34]	64.0	1032	464 (45)	Mean SBP: 140.0 mm Hg Mean DBP: 92.5 mm Hg
Zaleski et al (2019) [35]	52.3	24	13 (54)	Mean SBP: 136.2 mm Hg Mean DBP: 85.2 mm Hg Mean duration of hypertension: 6.2 years
Zha et al (2019) [36]	52.2	25	22 (88)	Mean SBP: 145.72 mm Hg Mean DBP: 90.57 mm Hg

^a^SBP: systolic blood pressure.

^b^PGHD: patient-generated health data.

^c^DBP: diastolic blood pressure.

^d^UC: usual care.

^e^ABPM: ambulatory blood pressure monitoring.

Table 2 (isolated-effect studies) and Multimedia Appendix 1 (Table S2; multicomponent studies) show the patient characteristics from the 49 studies. The mean age ranged from 49 to 73 years, and the percentage of females ranged from 5% to 88%. The mean baseline SBP was reported in 44 (90%) studies and ranged from 125 to 161 mm Hg. The mean baseline DBP was reported in 42 (86%) studies and ranged from 71 to 97 mm Hg. Only 3 (6%) studies were conducted in rural populations [25,37,38], whereas 24 (49%) were not of rural populations [22,23,26,29,30,35,36,39-62] and the other 22 (44%) did not specify.

Only 21 of the 49 (43%) studies reported health outcomes, which included mortality (n=3 studies, 6%), hospitalizations or emergency room visits (n=2 studies, 4%), QoL (n=13 studies, 26%), and AEs (n=13 studies, 26%). No studies reported other health outcomes related to hypertension, such as major adverse cardiovascular events. All studies reported SBP, DBP, or BP control.

### Device Characteristics

The included studies used 41 different BP monitoring devices (see specifics in Table 1). Of these, 34 (83%) were arm devices and 2 (5%) were wrist devices, and the wrist or arm was unclear in the other 5 (12%) studies. A total of 38 (93%) BP monitors were judged as similar to devices currently on the market from the corresponding manufacturer, 1 (2%) was judged as somewhat different, and 2 (5%) were of unknown similarity.

Regarding the transmission of data (eg, to a website, to study staff, or to health care providers), 19 of 49 (39%) studies used automatic transmission, 6 (12%) used manual data entry for transmission, 20 (41%) had no electronic data transmission, and the other 4 (8%) did not report whether or how data were transmitted.

### Isolated Effects on Health Outcomes

The isolated effects of a consumer BP monitor device on health outcomes were evaluated in 6 of the 49 (12%) studies. The consumer BP monitors examined included the iHealth BP 7 Wireless Wrist Monitor, Omron 637, Omron 773AC, Omron HEM-705 CP, Omron HEM-712C, and Omron M10-IT. Only 1 of the 6 (17%) studies reported mortality [22], 1 (17%) reported hospitalization [14], and the other 4 (67%) reported QoL [4,18,20,21,31-33,36].

- For mortality, Hebert et al [22] followed patients for 18 months and found that 8 deaths occurred in the 3 study groups (Omron HEM-712C BP monitor, Omron HEM-712C BP monitor plus nurse management, and usual care). Mortality rates did not differ significantly across the groups (group-specific rates were not reported).
- For hospitalizations, Bosworth et al [14] reported no statistically significant differences in hospitalization rates among the 4 study groups. The rates ranged from 19% to 23% (group-specific rates were not reported). The groups received Omron 773AC or 637 (depending on patient arm circumference) compared with usual care, behavioral management alone, or a combination of BP monitoring and behavioral management.
- For QoL, 3 of the 4 (75%) studies found no statistically significant differences between groups at follow-ups ranging from 3 to 12 months. To measure QoL, the studies used the Short Form Health Survey 36 (SF-36) [18], the Short Form Health Survey-12 [20,21], or the EQ-5D [4,31-33]. The fourth study [36] found that at both baseline and the 6-month follow-up, there was a statistically significant difference in SF-36 scores favoring the usual care group over the BP monitor group (suggesting a problem with randomization rather than an effect of the BP monitor).

### Isolated Effects on Surrogate Outcomes

Of the 49 studies, 18 (37%) [4,13-26,28-36] examined the isolated effects of consumer BP monitors on blood pressure. All evaluated the effects compared with usual care (ie, no BP monitor), except for 2 (11%) studies [25,29], each of which compared BP monitors with automatic data transmission with BP monitors without automatic transmission.

**Figure 2:**
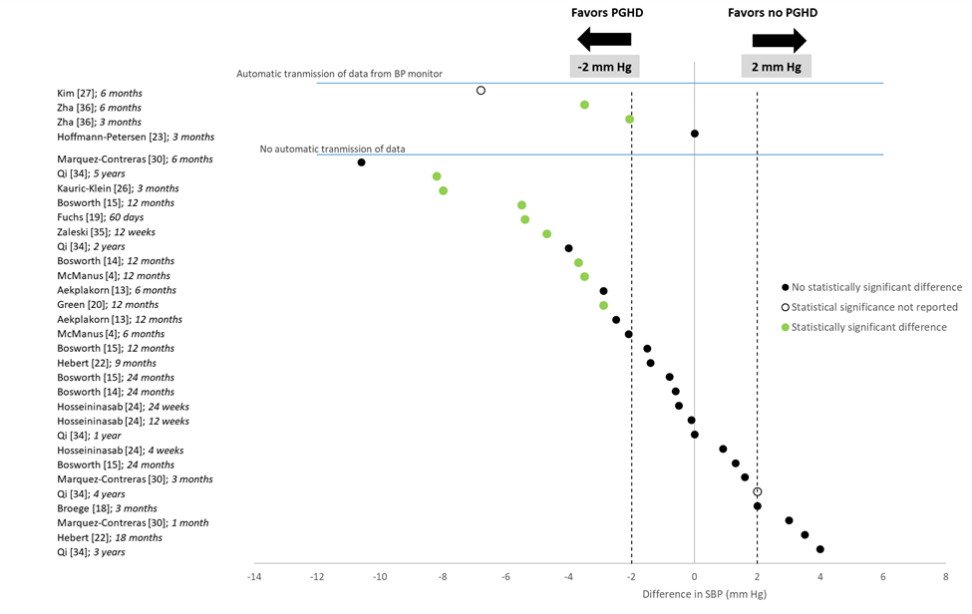
Systolic blood pressure (SBP) differences in studies of isolated effects of blood pressure (BP) monitors. PGHD: patient-generated health data.

All 16 studies on comparisons with usual care reported the effects of PGHD interventions on SBP (Figure 2). The top 4 points were from studies using automatic transmission of BP data, and the remaining 28 points were from studies that did not use automatic transmission. Six studies [4,15-17,19-21,26,31-34] found a statistically significant reduction in SBP favoring the BP monitoring group compared with the control group. However, the results were somewhat inconsistent. For example, Bosworth et al [15-17] found significant improvement only in non-White patients at 12 months; differences were not statistically significant for White patients at any time point or 24 months for any subgroup. The point estimates for SBP are shown in Figure 2, corresponding to 32 reported outcomes from 16 studies. Moreover, 4 of 32 (13%) SBP outcomes identified a reduction of 6 mm Hg or more favoring the consumer BP monitor group compared with usual care; 12 (38%) identified an SBP reduction between 2 mm Hg and 6 mm Hg favoring the consumer BP monitor, 10 (31%) identified SBP differences from −2 mm Hg to +2 mm Hg, and the remaining 3 (9%) found an SBP reduction ≥2 mm Hg favoring the usual care groups. Whether the BP monitor automatically transmitted data (comparing the top 4 points with the other points) did not appear to modify the effect on SBP.

**Figure 3:**
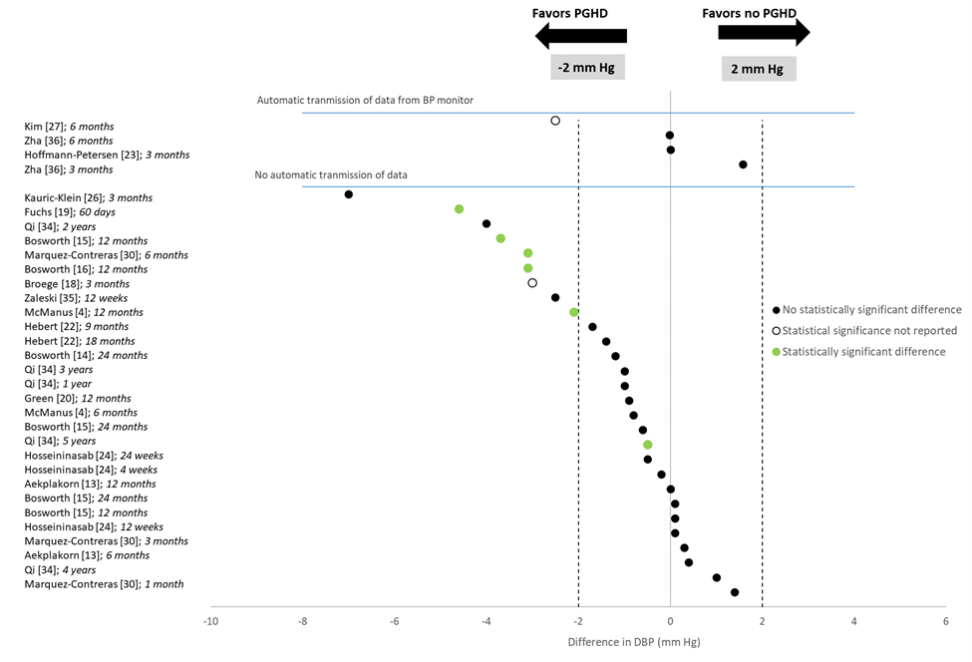
Diastolic blood pressure (DBP) differences in studies of isolated effects of blood pressure (BP) monitors. PGHD: patient-generated health data.

The overall findings for DBP were similar to those for SBP; 5 (31%) [4,15,19,30-34] studies found that consumer BP monitors significantly reduced DBP compared with controls. However, similar to SBP, the results were inconsistent, and statistical significance was found only for particular subgroups or time points in a study. The 32 point estimates for DBP are shown in Figure 3 (restricted to studies with usual care comparison groups). Of these, 1 (3%) identified a DBP reduction of 6 mm Hg or more favoring the consumer BP monitor, 9 (28%) identified a DBP reduction between 2 mm Hg and 6 mm Hg, favoring the consumer BP monitor, and the remaining 19 (59%) identified DBP differences from −2 mm Hg to +2 mm Hg. Whether the BP monitor automatically transmitted data did not appear to modify its effect on DBP.

Regarding the 2 studies examining the effect of data transmission (eg, BP monitor with vs without data transmission), Kaihara et al [25] found that data transmission resulted in an estimated 6 mm Hg lower SBP but no statistically significant effect on DBP. Lakshminarayan et al [29] found a statistically nonsignificant difference of 3.7 mm Hg in favor of data transmission and did not report data on DBP.

BP control was examined in 9 (15%) studies of the isolated effects of consumer BP monitors [13,14,19-23,27,28,30,34]. Most defined BP control as SBP <140 mm Hg and DBP <90 mm Hg, but 1 study [23] used <135/<85 mm Hg; 2 [14,23] studies included a separate definition of <130/80 mm Hg for patients with diabetes. Only 2 of the 9 (22%) studies [19,34] reported statistically significantly higher rates of BP control with BP monitors than with controls.

- Fuchs et al [19] found that at 60 days, the BP control rates measured in the office were similar for BP-monitored patients and usual care patients (43% and 41%, respectively), but for 24-hour BP, 32% of BP-monitored patients had BP control compared with only 16% of usual care patients;
- Qi et al [34] found that at 5 years, 85% of BP-monitored patients had BP control compared with 80% of usual care patients.

The remaining 7 (78%) studies found nonsignificant differences in BP control rates between BP-monitored and control patients.

### Isolated Effects on Process Outcomes

Of the 18 studies on isolated effects of BP monitors, 5 (28%) reported process outcomes, and the results were mixed. For medication prescribing, McManus et al [4,31-33] found that those in the BP monitor group were prescribed statistically significantly more antihypertensive drugs than those in the usual care group (difference 0.11; 95% CI 0.02-0.19), and 3 other studies found no statistically significant impact of BP monitoring on prescriptions.

- Hebert et al [22] reported that the percentage of patients who had no change in medications at 9 months was not statistically significantly different among those who had BP monitoring (44%) compared with the control group (38%).
- Hoffmann-Petersen et al [23] found that at baseline, 59% of the BP-monitored group and 61% of the control group did not receive any antihypertensive medication. At follow-up, these percentages were reduced to 23% in the BP-monitored group and 22% in the control group (not a significant difference).
- Aekplakorn et al [13] found that prescription of antihypertensive medications increased in both groups, but there were no significant between-group differences in drug items or drug classes (the authors did not report the number of prescriptions at follow-up).

However, these studies were not statistically powered to detect such effects, so they did not rule out the possibility of an impact on prescriptions.

In addition, Bosworth et al (2009) [14] found no between-group differences in the number of outpatient encounters (medians ranged from 13 to 15).

With regard to data transmission, 5 of the 18 (28%) studies used automatic data transmission, 2 (11%) used manual entry, 10 (31%) had no electronic data transmission, and 1 (3%) did not report whether or how data were transmitted. Of those using automatic data transmission, in Hoffmann-Petersen et al [23], data were transmitted using a Tunstall RTX3371 or Numera telehealth monitor to a study database or an electronic health record after BP measurements.

In Kaihara et al [25], the BP monitor wirelessly transmitted data to a study database over the internet.

In Kim et al [27], the BP monitor readings were wirelessly transmitted via the HealthCircles app on a smartphone to a website.

In Lakshminarayan et al [29], a smartphone transmitted daily BP measurements to a study database. Participants in the PGHD group transmitted data on an average of 89% of the study days and rated the ease of use of the system favorably.

In Zha et al [36], the wireless BP wrist monitor would transmit data to a website using the iHealth MyVitals app on a smartphone.

Of the 18 studies, 2 (18%) studies used manual data transmission [4,35]. In these 2 studies, participants sent BP readings via an SMS text message service or web-based form to a website [4] or entered their BP measurements on a BP-tracking website [35].

### Adverse Events

Of the 49 studies, 12 (24%) reported on AEs and generally found them to occur infrequently, and 4 [4,14,20,21,31-33,35] of the 18 (22%) studies on isolated effects of BP monitoring reported on AEs; 2 (17%) studies reported that no AEs occurred during the course of the study. A study [20,21] found that serious AEs, including nonfatal cardiovascular events, were rare and not substantially different between the BP monitoring and control groups. Another study [4,31-33] reported on various other AEs, including pain, fatigue, and dry mouth. Only dry mouth occurred significantly more frequently in the BP monitor group than in the usual care group. Of the 49 studies, 11 (22%) [4,14,20,21,31-33,38,44-46,50-56,60,63-74] reported on AEs in studies with multicomponent device groups. Only one of those studies [69-73] reported a significant increase of an AE, swelling of legs, in a multicomponent intervention group that included a BP monitor compared with usual care.

### Consumer Experience

Of the 49 studies, 26 (53%) reported the outcomes of consumer experience. Adherence to the use of BP monitors ranged from 38% to 89%, but device adherence had variable definitions. For example, Logan et al [47] defined adherence as a minimum of 8 readings per week. Zaleski et al [35] only determined whether patients said they were still monitoring their BP. Zha et al [36] measured adherence by dividing the number of received readings by expected readings.

Some studies reported that adherence declined throughout the study. For example, Bosworth et al [14] reported that during the first 2 months, 91% of those using a BP monitor were adherent, whereas 64% were adherent during the last 2 months. The studies also measured BP monitor use in various ways, including the total number of transmissions during the study and the average number of transmissions per week.

Studies measuring the ease of use or satisfaction with consumer BP monitors found favorable ratings. For example, Magid et al [49] reported that 68% of patients using the monitor found it very or extremely easy to use. Rifkin et al [75] reported that 96% of patients would continue to use the BP monitor.

Only 2 studies reported problems with BP monitors. Bosworth et al [63-65] found that 35 alerts were triggered by the monitoring system due to BP monitor problems, which represented 5% of the total alerts that occurred during the study. Lakshminarayan et al [29] found that some patients experienced issues with the BP monitor and the smartphone provided to transmit BP data, including an inability to hold a charge and difficulty using the phone app to see BP data.

### Multicomponent Effects

Of the 31 multicomponent studies [38-87], 11 (35%) examined the multicomponent effect of BP monitors on health outcomes, and all 31 evaluated multicomponent PGHD for surrogate outcomes including SBP, DBP, and BP control. These study designs did not permit any determination of the effectiveness specific to BP monitors.

### Risk of Bias

Of the 18 studies of isolated effects, we rated 6 (33%) as low risk of bias, 9 (50%) as moderate risk of bias, and 3 (17%) as high risk of bias. In contrast, of the 31 studies of multicomponent effects, we rated 6 (19%) as low risk of bias, 13 (42%) as moderate risk of bias, and 12 (39%) as high risk of bias. The full AHRQ report (in its Appendix Table C-26) contains the item-level and overall risk-of-bias ratings for each study [7].

## Discussion

### Principal Findings

This systematic review summarizes 49 comparative studies that used consumer BP monitors for hypertension management. However, the effects of these devices on health outcomes remain unclear. Only 18 studies were designed to isolate the BP monitor effect, and only 6 of these 18 (33%) studies reported any health outcome, such as mortality, hospitalization, and QoL. One study [36] found a statistically significant difference in QoL at follow-up favoring usual care over BP monitoring, but QoL also favored usual care at baseline (suggesting a problem in the randomization process). None of the 5 remaining studies found statistically significant effects on health outcomes, possibly because they were powered to detect differences in BP measurements and not necessarily differences in health outcomes. Many studies had only 6 months of follow-up, which may also explain the uncertain effect of BP monitors on health outcomes.

We found consistent benefits of BP monitoring on both surrogate outcomes, SBP and DBP. SBP reductions typical of included studies ranged between 2 and 4 mm Hg, and DBP reductions ranged from 1 to 3 mm Hg. It is unclear whether these modest changes in BP related to consumer BP monitors lead to lower risks of hypertension-related complications or mortality. Many factors may have potentially modified BP reduction in these studies. BP self-monitoring may support behavioral changes or reminder strategies to assist with lifestyle changes or medication adherence [2-4]. In addition, select BP monitors transmit data to health care providers and can improve BP control by facilitating timely recommendations from providers to patients to better manage their BP [87,88]. However, only 5 [23,25,27-29,36] of the 18 (27%) studies on isolated effects of BP monitors used automatic data transmission, and the effects on provider behavior change were rarely described among the included studies. This indicates that many studies did not use the advanced capabilities of modern BP monitors and may explain the unclear impact on health outcomes.

Most studies reported adherence to BP monitor use that ranged from 38% to 89%, but adherence was inconsistently measured. There was also a large gap between self-reported and measured adherence, such as a set number of recordings per week, as self-reported information is not always reliable. In addition, adherence can be affected by a variety of factors, such as daily access to the device, consumer comfort with the device, or self-motivation factors [89]. Spillover to other adherence factors, such as medication adherence or compliance with lifestyle behavior changes to manage hypertension, were not reported but may ultimately be a mechanism by which consumers of BP monitors improve their hypertension. Another consumer experience outcome, overall satisfaction, was reported as highly favorable among the included studies, thus validating the current rising consumer market for these devices.

Many studies evaluated multicomponent interventions, with BP monitors representing only 1 component, and did not separately evaluate the impact of the BP monitor. In our evidence base, only 18 of the 49 (37%) studies permitted such a direct assessment of BP monitor impact. Many PGHD technologies are intended to be used in combination with other interventions for chronic disease management, such as additional devices, exercise sessions, or health education sessions with medical personnel. These interventions may also influence outcomes; therefore, studies should be designed to measure the impact of isolated PGHD technology when added to other components.

### Strengths and Limitations

This systematic review has several strengths. To our knowledge, this is the first systematic review to synthesize the patient-centered health effects of consumer BP monitors for hypertension management, in addition to their effects on BP. We closely followed the PRISMA (Preferred Reporting Items for Systematic Reviews and Meta-Analyses) reporting standards and used robust AHRQ Evidence-based Practice Center systematic review methodology, including duplicate literature screening and data extraction. The findings of our review mirror those from 2 recent meta-analyses of systematic reviews of individual patient data [90,91] and contribute summary-level data on health effects as well as key data on medication management and consumer experience. Furthermore, in this review, we used device engineers to verify the consumer availability of BP monitors used in studies and their similarity to currently available models.

This systematic review has limitations related to both the review methodology and the generalizability of the available literature. We judged the overall risk of bias using an adapted tool designed for mobile apps in managing diabetes [8] and therefore may not have detected some biases. We did not assess the possibility of publication bias, which may be a key problem in studies funded by manufacturers of devices that collect PGHD. The included studies rarely provided sufficient detail to delineate the contributions of cointerventions to outcomes, particularly those related to changes in BP. This limits the generalizability of our findings to patients with limited access to care or underserved patient populations. This may also further limit the confidence in the validity of our findings not otherwise captured in our risk-of-bias assessment. Studies with *usual care* groups often provided few details about what happened with these patients, which may potentially explain the wide variation in BP results among studies. The inclusion criteria of multiple studies were specific to consumers who had access to and familiarity with technology, which could include using the internet, smartphones or computers, arm or wrist devices, or access to electricity. Less technically adept consumers may not experience the same benefits as those enrolled in these studies. In addition, only 3 [25,37,38] of the 49 (6%) studies focused on rural populations, suggesting that these populations are underrepresented. Only 19 of the 49 (39%) studies used automatic data transmission from PGHD devices to health care providers.

Future studies are needed to examine the effectiveness of BP monitors that transmit data to health care providers (which are then used to inform medical decisions). Additional data from implementation studies may help determine which components are critical for sustained BP improvement, which in turn may improve prescription decisions by clinicians and coverage decisions by policy makers. In addition, challenges related to data accuracy, interoperability, privacy, and security should be explored as this field continues to grow.

## Appendix

Multimedia Appendix 1 Tables showing general characteristics and patients characteristics of the 31 multicomponent studies.

## Abbreviations

AE: adverse event
AHRQ: Agency for Healthcare Research and Quality
BP: blood pressure
DBP: diastolic blood pressure
FDA: Food and Drug Administration
PGHD: patient-generated health data
PRISMA: Preferred Reporting Items for Systematic Reviews and Meta-Analyses
QoL: quality of life
SBP: systolic blood pressure
SF-36: Short Form Health Survey 36
