# Supplementary material for: Consumer Devices for Patient-Generated Health Data Using Blood Pressure Monitors for Managing Hypertension: Systematic Review
Source: JMIR Mhealth Uhealth. 2022 May 2;10(5):e33261. doi: 10.2196/33261 (PMC9112087; doi:10.2196/33261)
Supplement: Multimedia Appendix 1 [file mhealth_v10i5e33261_app1.docx]

# **Appendix Tables**

Appendix Table S-1. General Characteristics in Multicomponent Studies

| Study | Design | Country | N at Baseline | Study Duration | Study groups (BP monitor manufacturer/model) | Outcomes |
| --- | --- | --- | --- | --- | --- | --- |
| Bernocchi 2014[38] | NRS | Italy | 168 | Mean of 80 days | Combination (Structured physician-directed, nurse-managed telemonitoring + BP telemonitoring [A&D UA-767 Plus])  Usual care | Surrogate (SBP, DBP, BP control) |
| Bosworth et al. (2011)[39-41] | RCT | USA | 591 | 18 months | Combination (PGHD UA-767PC wireless home BP monitor + behavioral management)  Combination (PGHD UA-767PC wireless home BP monitor + medication management)  Combination (PGHD UA-767PC wireless home BP monitor + behavioral management + medication management)  Usual care | Surrogate (SBP, DBP, BP control)  Adverse events  Consumer experience |
| Bove et al. (2013)[42] | RCT | USA | 241 | 6 months | PGHD (BP+ pedometer + scale)  Usual care | Surrogate (SBP, DBP, BP control)  Consumer experience |
| Dorough et al. (2014)[43] | RCT | USA | 23 | 10 weeks | DASH 2 wellness plus  DASH 2 wellness only | Surrogate (SBP) |
| Earle et al. (2010)[44] Istepanian, 2009[45] | RCT | UK | 137 | 6 months | Combination (One Touch Ultra Glucose Meter +BP monitoring using UA-767BT, treatment management)  Usual care | Surrogate (SBP); Consumer experience |
| Green et al. (2014)[46] | RCT | USA | 101 | 26 weeks | Combination (Web dietician + scale (device NR), + pedometer (device NR) + a home BP monitor [Omron 711-DLX])  Usual care | Surrogate (SBP, DBP)  Consumer experience |
| Halme et al. (2005)[47] | RCT | Finland | 232 | 6 months | Combination (PGHD Omron M4 + Physician management)  Usual care | Surrogate (SBP, DBP, BP control) |
| He et al. (2017)[48] | RCT | Argentina | 1432 | 18 months | Combination (health coaching + PGHD using Omron HEM-737 automatic home BP monitor +BP audit)  Usual care | Surrogate (SBP, DBP, BP control) |
| Kao et al. (2019)[49] | RCT | Taiwan | 222 | 6 months | Combination (PGHD Omron Colin JPN1 + health education)  Usual care | Health (QOL)  Surrogate (SBP, DBP)  Process |
| Kerry et al. (2013)[50-52] | RCT | UK | 381 | 52 weeks | Combination (PGHD Omron M6 + nurse intervention)  Usual care | Health (QOL)  Surrogate (SBP, DBP)  Adverse events  Process  Consumer experience |
| Kim et al. (2015)[53] | RCT | South Korea | 374 | 6 months | PGHD (UA 767PBT)  Combination (PGHD LG Smartcare System + remote monitoring by physician, nurse, nutritionist, and exercise trainer)  Combination (PGHD LG Smartcare System + remote monitoring by physician, nurse, nutritionist, and exercise trainer + remote physician care) | Surrogate (SBP, DBP)  Consumer experience |
| Klarskov et al. (2018)[54] | RCT | Denmark | 1048 | 12 months | Combination (PGHD Microlife BP3AC1 + intensive monitoring)  Usual care | Surrogate (SBP, DBP) |
| Logan et al. (2012)[55] | RCT | Canada | 110 | 1 year | Combination (PGHD A&D Life Source UA-767 + Self-Care Support System)  BP monitor without Bluetooth transmission capability | Surrogate (SBP, DBP, BP control)  Consumer experience |
| Magid et al. (2011)[56] | RCT | USA | 283 | 6 months | Combination (PGHD LifeSource UA-767 BP + BP Reporting +Education, Pharmacist Management)  Usual care | Surrogate (SBP, DBP, BP control) |
| Magid et al. (2013)[57] | RCT | USA | 348 | 6 months | Combination (PGHD Omron HEM 790 IT + Pharmacist care)  Usual care | Health (hospitalization)  Surrogate (SBP, DBP, BP control)  Process  Consumer experience |
| Margolis et al. (2013)[58-64] | RCT | USA | 450 | 18 months | Combination (PGHD A&D Medical 767PC automated oscillometric arm BP monitor + Pharmacist Care)  Usual care | Health (QOL)  Surrogate (SBP, DBP, BP control)  Process  Adverse events  Consumer experience |
| McKinstry et al. (2013)[65-67] | RCT | UK | 401 | 6 months | Combination (PGHD Stabil-O-graph mobile + Text/email support)  Usual care | Health (mortality, hospitalization; QOL)  Surrogate (SBP, DBP)  Process  Adverse events  Consumer experience |
| McManus et al. (2010)[68-72] | RCT | UK | 480 | 12 months | Combination (PGHD Omron 705IT BP monitor + medication management)  Usual care | Health (QOL)  Surrogate (SBP, DBP)  Adverse events  Consumer experience |
| McManus et al. (2014)[73] | RCT | UK | 555 | 12 months | Combination (PGHD Microlife Watch BP Home + individualized self-titration algorithm)  Usual care | Health (QOL)  Surrogate (SBP, DBP)  Process  Adverse events |
| Mehos et al. (2000)[74] | RCT | USA | 41 | 6 months | Combination (PGHD A&D UA-702 manual electronic blood pressure monitor + pharmacist care)  Usual care | Health (QOL)  Surrogate (SBP, DBP, BP control)  Process  Consumer experience |
| Mendelson et al. (2014)[75] | RCT | France | 107 | 17 weeks | Combination (PGHD Telemedicine Omron 705CP + Self-care messaging)  Usual care | Health (QOL)  Surrogate (SBP, DBP) |
| Neumann et al. (2011)[76, 77] | RCT | Germany | 60 | 3 months | Combination (PGHD IEM Stabil-O-Graph automatic BP monitor + Physician management)  Usual care | Surrogate (SBP, DBP, BP control) |
| Niiranen et al. (2014)[78] | RCT | Finland | 220 | 12 months | Combination (PGHD Omron HEM-722C + behavioral + pharmaceutical) vs Usual care | Surrogate (SBP, DBP, BP control) |
| Ogedegbe et al. (2014)[79] | RCT | USA | 1039 | 12 months | Combination (PGHD Microlife model BP 3AC1-1PC + health education + counseling)  Usual care | Health (mortality)  Surrogate (SBP, DBP, BP control)  Process  Adverse events  Consumer experience |
| Petrella et al. (2014)[80] | NRS | Canada | 149 | 52 weeks | Combination (Exercise + Healthanywhere app + BP monitor (A&D UA-767PBT) + glucometer (Lifescan One Touch Ultra2)  Active control (Exercise) | Surrogate (SBP, DBP, BP control)  Adverse events Consumer experience |
| Rifkin et al. (2013)[81] | RCT | USA | 43 | 6 months | Combination (PGHD A&D Medical UA-767PBT + Physician management)  Usual care | Surrogate (SBP, DBP); Consumer experience |
| Rogers et al. (2001)[82] | RCT | USA | 121 | Median  11 weeks | Combination (PGHD Welch Allyn Model 52500 BP + Physician management)  Usual care | Surrogate (SBP, DBP, BP control) |
| Sarfo et al. (2018)[83, 84] | RCT | Ghana | 60 | 9 months | Combination (PGHD UA-767Plus BT BP + motivational messaging)  Usual care | Surrogate (SBP, DBP, BP control)  Consumer experience |
| Stewart et al. (2014)[85] | RCT | Australia | 395 | 6 months | Combination (Pharmacist Care + Omron HEM-790IT BP monitor + reminders)  Usual care | Surrogate (SBP, DBP) |
| Yoo et al. (2009)[86] | RCT | South Korea | 111 | 13 weeks | Combination (Anycheck glucose monitor + Omron T5M BP monitor + HD308 scale)  Usual care | Surrogate (SBP, DBP)  Consumer experience |
| Zarnke et al. (1997)[87] | RCT | Canada | 31 | 8 weeks | Combination (PGHD Omron Marshall 85 oscillometric sphygmomanometer + treatment management)  Usual care | Health (QOL)  Surrogate (SBP, DBP, BP control)  Process |

ABPM: ambulatory blood pressure monitoring; BP: blood pressure; CPAP: continuous positive airway pressure; DBP: diastolic blood pressure; HBPM: home blood pressure monitoring; mmHg: millimeters of mercury; NR: not reported; NRS: non-randomized study; PGHD: patient generated health data; QOL: quality of life; RCT: randomized controlled trial; SBP: systolic blood pressure; SD: standard deviation; UC: usual care; UK: United Kingdom; USA: United States of America

Appendix Table S-2. Patient Characteristics in Multicomponent Studies

| Study | Mean Age | % Female | Baseline disease severity |
| --- | --- | --- | --- |
| Bernocchi et al. (2014)[38] | 59.4 | 48% | Mean SBP: 154.7 mmHg  Mean DBP: 86.0 mmHg |
| Bosworth et al. (2011)[39-41] | 64 | 8% | Mean SBP: 129 mmHg  Mean DBP: 77 mmHg |
| Bove et al. (2013)[42] | 59.6 | 65% | Mean SBP UC: 154.4 (SD: 16.3), PGHD: 155.9 (SD: 13.7)  Mean DBP UC: 87.6 (SD: 10.9), PGHD: 88.9 (SD: 11.2) |
| Dorough et al. (2014)[43] | 54 | 70% | NR |
| Earle et al. (2010)[44] Istepanian 2009[45] | 58.4 | NR | Mean SBP: 131.1 mmHg  Mean DBP: 76.8 mmHg |
| Green et al. (2014)[46] | 57 | 42% | NR |
| Halme et al. (2005)[47] | 57.3 | 67.2% | Mean office SBP:  159.3 mmHg  Mean office DBP:  94.4 mmHg |
| He et al. (2017)[48] | 55.8 | 53.0% | Mean SBP: 150.8 mmHg  Mean DBP: 91.2 mmHg |
| Kao et al. (2019)[49] | 62.7 (SD: 9.3) | 49% | Mean SBP: 143.2 (SD: 13.6) mmHg  Mean DBP: 84.2 (SD: 10.8) mmHg  Mean duration of hypertension: 6 years |
| Kerry et al. (2013)[50-52] | 72 | 43% | Mean SBP: 138 mmHg  Mean DBP: 74 mmHg |
| Kim et al. (2015)[53] | 57.1 | 42% | Mean SBP: PGHD: 143.2 (SD: 13) mmHg, PGHD + remote monitoring: 142.9 (SD: 14.5) mmHg, PGHD + remote monitoring + remote care: 143.1 (SD: 14.7) mmHg  Mean duration of hypertension: PGHD: 8.4 (SD: 6.8) years, PGHD + remote monitoring: 8.9 (SD: 8) years, PGHD + remote monitoring + remote care: 7.2 (SD: 6.4) years |
| Klarskov et al. (2018)[54] | 61.8 | 48% | Mean daytime systolic ABPM: 137.7 mmHg  Mean daytime diastolic ABPM: 82.1 mmHg  Mean nighttime systolic ABPM: 122.8 mmHg  Mean nighttime diastolic ABPM: 70.4 mmHg |
| Logan et al. (2012)[55] | 62.9 | 44% | Mean 24-hour SBP: 139.7 mmHg  Mean 24-hour DBP: 74.7 mmHg |
| Magid et al. (2011)[56] | 62 | 33% | Mean SBP: 147.1 mmHg  Mean DBP: 87.3 mmHg |
| Magid et al. (2013)[57] | 59.5 | 39.7% | Mean SBP UC: 145.5 (SD: 14.5) mmHg, PGHD: 148.8 (SD: 16.2) mmHg  Mean DBP UC: 88.0 (SD: 9.9) mmHg, PGHD: 89.6 (SD: 10.2) mmHg |
| Margolis et al. (2013)[58-64] | 61.1 | 44.7% | Mean SBP: 147.9 mmHg Mean DBP: 84.7 mmHg |
| McKinstry et al. (2013)[65-67] | 60.6 | 40.5% | Mean clinic BP: SBP PGHD: 152.9 (SD: 15.1), UC: 152.4 (SD: 14.3),  DBP PGHD: 92.1 (SD: 11.5), UC: 89.9 (SD: 11.3)  Mean daytime ambulatory BP: SBP PGHD: 146.2 (SD: 10.6) and UC: 146.2 (SD: 10.5) and DBP PGHD: 87.1 (SD: 10.0), UC: 85.4 (SD: 9.6) |
| McManus et al. (2010)[68-72] | 66.4 | 53% | Mean SBP: 152.0 mmHg  Mean DBP: 84.8 mmHg |
| McManus et al. (2014)[73] | 69.5 | 40% | Mean DBP: 143.4 mmHg  Mean SBP: 80.1 mmHg |
| Mehos et al. (2000)[74] | 57.6 (control) vs. 60.0 (PGHD) | 69.4% | Mean SBP: Control: 153.9 (SD: 14.6) mmHg, PGHD: 157.9 (SD 16.4) mmHg  Mean DBP: Control: 89.6 (SD: 9.8) mmHg, PGHD: 91.1 (SD: 10.8) mmHg  Mean arterial pressure: Control: 111.0 (SD: 6.4) mmHg, PGHD: 113.4 (SD: 8.0) mmHg |
| Mendelson et al. (2014)[75] | 63 | 17% | Mean SBP: 139 mmHg  Mean DBP: 81 mmHg |
| Neumann et al. (2011)[76] Neumann, 2015[77] | 55.5 | 52% | Mean 24-hour systolic ABPM: 143.4 mmHg |
| Niiranen et al. (2014)[78] | 62.2 | 50% | Mean SBP: 147 mmHg  Mean DBP: 87 mmHg |
| Ogedegbe et al. (2014)[79] | 56.5 (SD: 12.1) | 72% | Mean SBP: 151 (SD: 17) mmHg  Mean DBP: 91 (SD: 11) mmHg |
| Petrella et al. (2014)[80] | 57 | 74% | Mean SBP: 141 mmHg  Mean DBP: 85 mmHg |
| Rifkin et al. (2013)[81] | 68.3 | 5% | Mean SBP: 148 mmHg  Mean DBP: 79 mmHg |
| Rogers et al. (2001)[82] | 61.5 | 50% | NR |
| Sarfo et al. (2018)[83] Sarfo, 2018[84] | 55.1 | 35% | SBP Mean: 143.8  DBP Mean: 90.5  Mean number of antihypertensive medications: 2.7 |
| Stewart et al. (2014)[85] | 66.7 | 49% | Mean SBP: 141.0 mmHg  Mean DBP: 83.8 mmHg |
| Yoo et al. (2009)[86] | 58 | 41% | NR |
| Zarnke et al. (1997)[87] | 55 | 65% | Mean arterial BP: 97 mmHg |

ABPM: ambulatory blood pressure monitoring; ACC/AHA: American College of Cardiology/American Heart Association; BMI: body mass index; BP: blood pressure; CKD: chronic kidney disease; CPAP: continuous positive airway pressure; CT: computed tomography; CVD: cardiovascular disease; DBP: diastolic blood pressure; DM: diabetes mellitus;
ECG: electrocardiogram; HbA1c: hemoglobin A1c; HBPM: home blood pressure monitoring; HDL: high-density lipoproteins; ICD: International Classification of Diseases; JNC: Joint National Committee; mmHg: millimeters of mercury; NR: not reported; NRS: non-randomized study; PGHD: patient-generated health data; RCT: randomized controlled trial;
SBP: systolic blood pressure; SCORE: Systematic Coronary Risk Evaluation; SD: standard deviation; UC: usual care; USB: Universal Serial Bus
